# Performance of non-invasive prenatal testing for foetal chromosomal abnormalities in 1048 twin pregnancies

**DOI:** 10.1186/s13039-021-00551-4

**Published:** 2021-06-30

**Authors:** Yuan Cheng, Xinran Lu, Junxiang Tang, Jingran Li, Yuxiu Sun, Chaohong Wang, Jiansheng Zhu

**Affiliations:** 1 Affiliated Maternity and Child Health Hospital of Anhui Medical University, Hefei, China; 2 Maternity and Child Health Hospital of Anhui Province, Hefei, China

**Keywords:** Non-invasive prenatal testing, Chromosomal aneuploidy, Microdeletion, Microduplication, Twin pregnancy

## Abstract

**Objective:**

To investigate the clinical value of non-invasive prenatal testing (NIPT) to screen for chromosomal abnormalities in twin pregnancies and to provide further data on NIPT manifestations in twin pregnancies.

**Materials and methods:**

In a 4-year period, 1048 women with twin pregnancies were voluntarily prospectively tested by NIPT to screen for chromosomal abnormalities by sequencing cell-free foetal DNA (cffDNA) in maternal plasma. Positive NIPT results were confirmed by karyotyping, while negative results were followed up 42 days after delivery.

**Results:**

Thirteen women had positive NIPT results as follows: 2 cases of trisomy 21 (T21), 1 of trisomy 18 (T18), 7 of sex chromosome aneuploidy (SCA), 1 of microdeletion, and 2 of microduplication. Of these 13 cases, 2 were true-positive cases confirmed by foetal karyotype analysis, namely, 1 case of T21 and 1 of microdeletion. Furthermore, the remaining 11 high-risk pregnant women were confirmed as false positive by foetal karyotyping. Thus, the combined positive predictive value (PPV) of NIPT screening for chromosomal abnormalities in twin pregnancies was 15.4% (2/13). There were no false-negative case via our follow-up results.

**Conclusion:**

Safe and rapid NIPT has a certain clinical application value; however, the PPV is limited, and the screening efficiency is not stable. Careful use should be made in the screening of chromosomal abnormalities in twin pregnancies.

## Background

Non-invasive prenatal testing (NIPT) is a new method for screening foetal chromosomal abnormalities based on cffDNA in the peripheral blood of pregnant women and has been widely used in clinical practice. A large number of studies at home and abroad have shown that this technology has a high sensitivity and specificity for screening chromosomal aneuploidies in singleton pregnancies [1, 2]. However, there are few early studies on the applicability and relevance of this technique in pregnant women with twin pregnancies. In recent years, due to the rapid development of assisted reproductive technology, the rate of twin pregnancies in many countries in general has increased [3–5].

First, studies have shown that the risk of chromosomal abnormalities and serious complications in twin pregnancies is significantly higher than that in singleton pregnancies [6]. Second, the effectiveness of traditional serological screening methods in twin pregnancies is significantly decreased [7], and the use of the invasive diagnostic methods in twin pregnancies has the disadvantages of a difficult operation, and the potential for abortion and infection [8]. Therefore, a suitable prenatal screening method in twin pregnancies is urgently needed. Third, although sporadic studies have shown that NIPT has a certain screening efficacy in twin pregnancies [9], there is still no uniform conclusion. Fourth and most importantly, the current NIPT studies in twin pregnancies are focused mainly on common trisomy, and the use of NIPT in detecting sex chromosome diseases, microdeletions and microduplications has not been systematically studied. This study explored the feasibility of this technique in screening for chromosomal abnormalities in twin pregnancies and provided data-related information for such studies.

## Materials and methods

### Subjects

This was a prospective clinical study of 1048 twin pregnancies; the pregnant women underwent NIPT at the Maternity and Child Health Hospital of Anhui Province from March 1, 2016, to September 1, 2020. The inclusion criteria were as follows: (1) twin pregnancy confirmed by B ultrasound; (2) strong desire for NIPT; and (3) gestational age ≥ 12 weeks. The exclusion criteria were as follows: (1) pregnant women who received allogeneic blood transfusion, transplantation surgery, cell therapy or other possible interference with NIPT results and (2) definite chromosomal abnormalities in one member of the couple.

Before examination, all the pregnant women received professional and detailed genetic counselling regarding the NIPT screening target diseases, purpose, significance, accuracy, limitations, and other screening and diagnosis programmes to help them make decisions while fully informed and to provide informed consent. This study was approved by the Ethics Committee of Anhui Medical University. All the pregnant women and their families signed the application form and provided informed consent for prenatal testing of cffDNA from the peripheral blood of the pregnant women.

### DNA sequencing and bioinformatics analysis of maternal plasma

After the blood collection staff carefully checked each pregnant woman's information, sterile EDTA anticoagulant tubes were used to collect 5 mL peripheral blood from each woman. Blood samples were collected and sent to the high-throughput sequencing laboratory of Maternity and Child Health Hospital of Anhui Province at 4 °C. Plasma was isolated within 6 h with a two-step centrifugation protocol. The DNA was extracted by magnetic bead adsorption extraction method using QIAamp Circulating Nucleic Acid Kit (Qiagen). The high-throughput sequencing system Illumina NextSeq -550 AR was used for library construction, bridging PCR amplification, high-throughput sequencing and data analysis. The sequencing results were compared with the reference values of the normal human genome (hg19) [10]. The foetal score threshold for the pregnant women was set to 4%. The NIPT results are expressed as the Z-value risk score: a score ≥ 3 or ≤ − 3 was considered high risk, and a score > − 3 to < 3 was considered low risk. Experimental operations were conducted by professional laboratory personnel with theoretical knowledge and operational skills in molecular biology. All the experimental procedures were carried out after double checking the relevant information.

### Verification of the NIPT results

The pregnant women with low-risk NIPT results were informed of the importance of regular maternity examinations later in pregnancy, especially at 20–24 weeks of pregnancy, when the B ultrasound examination of excluded deformity is performed. The pregnant women with high-risk NIPT results received further counselling by clinical geneticists regarding the prenatal diagnosis. The pregnant women were informed of the necessity, risk rate and limitations of invasive prenatal diagnosis. With the informed consent of the patient, 20 mL amniotic fluid from the two foetuses was extracted by professional medical personnel under the guidance of B ultrasound for cell culture and karyotype analysis. After the foetal specimens were cultured, routine chromosome preparation and G banding were performed; specialists manually counted 30 chromosome karyotypes and analysed 5 chromosome karyotypes at the level of 400 bands. If the karyotype was abnormal, counting was repeated. The karyotype was described according to the “International System for Human Cytogenetic Nomenclature, ISCN2016”. If microdeletion or microduplication was found by NIPT, additional karyotype and microarray analysis was recommended. No abnormal findings during postpartum neonatal follow-up were defined as a negative result.

### Follow-up of pregnancy outcomes

All the pregnant women were followed up by telephone or electronic case record systems. Follow-up included pregnancy outcome, physical examination of the new-born and clinical and/or genetic diagnosis of any abnormalities.

## Results

### Maternal characteristics

A total of 1048 maternal blood samples from twin pregnancies were collected in the Maternity and Child Health Hospital of Anhui Province from March 1, 2016, to September 1, 2020. The basic characteristics of the women with twin pregnancies are shown in Table 1. The maternal age ranged from 18 to 48 years with a median age of 29 years, and 12.5% (131/1048) were 35 years old or older. The median gestational age at the time of blood collection was 16 weeks. For this study, there were 9 cases (0.9%) of monochorionic monoamniotic (MCMA) twins, 154 cases (14.7%) of monochorionic diamniotic (MCDA) twins, and 879 cases (83.9%) of dichorionic diamniotic (DCDA) twins. The chorionic characteristics were unknown in 6 cases (0.6%), and there were 0 cases (0%) of conjoined twins. Of all the twin pregnancies in this study, 730 (69.7%) were naturally conceived.

**Table 1:** Basic characteristics of the 1048 twin pregnancies

Characteristic	Number of cases (n, %)
*Maternal age*	
Median (years)	29
Range (years)	18–48
18–24 years	130 (12.4%)
25–29 years	455 (43.4%)
30–34 years	332 (31.7%)
≥ 35 years	131 (12.5%)
*Gestational age*	
Median (weeks)	16
Range (weeks)	12–28
12–15 weeks	322 (30.7%)
16–19 weeks	670 (63.9%)
20–23 weeks	46 (4.4%)
24–27 weeks	9 (0.9%)
≥ 28 weeks	1 (0.1%)
*Chorionicity*	
MCMA	9(0.9%)
MCDA	154 (14.7%)
DCDA	879 (83.9%)
Unknown chorionicity	6(0.6%)
Conjoined twins	0 (0.0%)
*Type of pregnancy*	
Assisted pregnancy	318 (30.3%)
Natural pregnancy	730 (69.7%)

Totals may not be 100% because of rounding

MCMA, monochorionic monoamniotic twin; MCDA, monochorionic diamniotic twin; DCDA, dichorionic diamniotic twin

### NIPT-positive cases

Thirteen pregnant women with twin pregnancies had NIPT-positive results.There were 2 cases of trisomy 21 (T21), 1 of trisomy 18 (T18), 7 of sex chromosome aneuploidy (SCA) (45,X: 5; 47,XXX: 1; 47,XXY: 1), 1 of microdeletion, and 2 of microduplication. No trisomy 13 (T13) was detected (Table 2). The remaining 1035 pregnant women had NIPT-negative results.

**Table 2:** The coincidence rate of each abnormal chromosome detected by NIPT

Abnormal chromosome	NIPT high-risk	Coincidence rate of prenatal diagnosis
T21	2	1/2
T18	1	0/1
T13	–	–
45,X	5	0/5
47,XXX	1	0/1
47,XXY	1	0/1
Microdeletion	1	1/1
Microduplication	2	0/2
Total	13	2/13

### Karyotype analysis results

The clinical details of the 13 women with high-risk twin pregnancies are summarized in Table 3. All of these women underwent an invasive prenatal diagnosis procedure and confirmation of the foetal karyotype after being informed of the process; they received genetic counselling by clinical geneticists. Microarray analysis of the microdeletions or microduplications was performed. The coincidence rate of each abnormal chromosome detected by NIPT is summarized in Table 2.

**Table 3:** Detailed information on the thirteen twin pregnancies with NIPT-positive results

Case	Maternal age (years)	Gestational age (weeks)	Placentation	Conception	NIPT result	Karyotyping	Clinical outcome
1	36	13 + 6	DCDA	NP	T21 high risk	N/T21	Reduction
2	30	17 + 0	DCDA	NP	T21 high risk	N/N	Natural birth
3	29	17 + 1	MCDA	NP	T18 high risk	N/N	Natural birth
4	26	18 + 4	DCDA	NP	Monosomy X	N/N	Natural birth
5	30	17 + 3	DCDA	NP	Monosomy X	N/N	Natural birth
6	26	18 + 4	DCDA	NP	Monosomy X	N/N	Caesarean
7	39	21 + 4	DCDA	NP	Monosomy X	N/N	Caesarean
8	32	16 + 5	DCDA	ART	Monosomy X	N/N	Caesarean
9	26	17 + 6	DCDA	NP	47,XXX	N/N	Natural birth
10	28	18 + 0	DCDA	NP	47,XXY	N/N	Caesarean
11	39	15 + 1	DCDA	NP	del (15) (q11.2q13.1), 6.13 Mb	N/Microdeletion	Reduction
12	29	17 + 2	DCDA	NP	dup (13) (q31.1q31.1), 2.37 Mb	N/N	Caesarean
13	29	14 + 4	DCDA	ART	dup (16) (p12.3p11.2), 16.38 Mb	N/N	Caesarean

NP, natural pregnancy; ART, artificial reproductive technology; N, normal

There were 2 true-positive cases (case 1 and case 11) in the high-risk twin pregnancies, including 1 case of T21 and 1 of microdeletion (Table 3). One of the foetuses of case 1 was T21, and the other foetus was euploid. Microarray analysis of case 11 was abnormal, which was consistent with the NIPT results. There was a copy number heterozygosity deletion in the long arm of chromosome 15, and the region size was 6.13 Mb. Karyotyping of case 2, case 3, case 4, case 5,case 6, case 7, case 8, case 9, case 10, case 12 and case 13 was normal, which was discordant with the NIPT results.

### Follow-up investigations

Based on the NIPT and karyotyping results, a foetal reduction operation of the abnormal foetus was performed in the true-positive cases of T21 and microdeletion. The 11 false-positive cases decided to continue their pregnancies, and eventually all gave birth to twins with a normal phenotype.

Among the 1035 women with NIPT-negative results, 1016 (98.2%) had follow-up results, and 19 (1.8%) were lost to follow-up (Table 4). A total of 977 pregnant women (94.4%) gave birth to twins with apparently normal phenotypes. Twenty-eight women (2.7%) were reported to have adverse pregnancy outcomes with no typical phenotype of chromosomal disease or mental retardation based on follow-up by telephone or through our electronic case record system. Another 11 women had unexplained miscarriages and embryonic arrest. No false-negative results were reported via our follow-up investigations.

**Table 4:** Outcomes of the 1035 twin pregnancies with NIPT-negative results

Clinical outcomes of NIPT negative cases (n = 1035)	Number of cases (n, %)
Normal twin birth	977 (94.4%)
Abnormal twin birth with no typical phenotype of chromosomal disease or mental retardation	28 (2.7%)
Birth defects (polycystic kidney, hearing impairment, umbilical hernia, etc.)	15 (1.4%)
One stillbirth foetus (preterm birth, intrauterine hypoxia, low body weight, etc.)	6 (0.6%)
Miscarriage (intrauterine infection, cervical incompetence, twin-twin transfusion syndrome, etc.)	7 (0.7%)
Unexplained miscarriage and embryonic arrest	11 (1.1%)
No follow-up because of loss of contact	19 (1.8%)
Total	1035 (100%)

### The performance of NIPT

One case of T21 and one case of chromosomal microdeletion were identified by NIPT in this study, and there were eleven cases of false-positive results. As a result, the combined positive predictive value (PPV) of NIPT screening for chromosomal abnormalities in twin pregnancies was 15.4% (2/13), the PPV of screening common trisomy was 33.3% (1/3), the PPV of screening T21 was 50% (1/2), and the PPV of screening chromosomal microdeletion and microduplication was 33.3% (1/3). The clinical manifestations of sex chromosomal diseases are varied, with the exception of Turner syndrome (45, X), most of these diseases have no obvious clinical manifestations in the infancy stage. And patients do not seek medical attention until adolescence because of developmental disorders or abnormal secondary sexual characteristics. Hence, the sensitivity and specificity of NIPT cannot be calculated, although none of the neonates in the study showed any symptoms or signs suggestive of chromosomal abnormalities.

## Discussion

The superior performance of NIPT in singleton pregnancies is recognized as an almost perfect screening method [1, 2], which makes the medical community and the public hopeful that the test technology can be equally applicable for pregnant women with twin pregnancies. At present, sporadic studies have shown the possibility of using NIPT in twin pregnancies. The results of studies indicating the efficacy of NIPT in twin pregnancies are summarized in Table 5. However, related studies are limited, and analysis of data on twin pregnancies is still lacking and controversial. More importantly, the current studies of NIPT in twin pregnancies are aimed mainly at T21, T18 and T13, and the use of NIPT for sex chromosome diseases, microdeletions, and microduplications has not been systematically studied. This is the first prospective study in which NIPT simultaneously detected autosomes and sex chromosome aneuploidies, microdeletions and microduplications as well in twin pregnancies.

**Table 5:** Studies reporting on screening for chromosomal abnormalities using cell-free DNA in twin pregnancies

Trial	Chromosomal abnormality	n	T 21	T 18	T 13	SCA	Micro deletion	Micro duplication	Population	False positive (n)	Sensitivity for T21 (%)
Tan et al. [4]	T21	565	4	–	–	–	–	–	Mixture	0	100
Fosler et al. [11]	T21, T18	487	6	0	–	–	–	–	Mixture	1	100
Le Conte et al. [12]	T21, T18	492	3	1	–	–	–	–	Mixture	1	100
Yang et al. [13]	T21, T18, T7, 47,XXX	432	4	1	–	0	–	–	Mixture	2	100
Yu et al. [14]	T21, T18, T13	1157	16	4	1	–	–	–	Mixture	0	100
He et al. [15]	T21	141	1	–	–	–	–	–	Mixture	0	100
Motevasselian et al. [16]	T21, T18, T13	356	2	2	1	–	–	–	High risk	0	100
Khalil et al. [17]	T21, T18, T13	958	13	1	1	–	–	–	Mixture	1	100
Our study	T21, T18, 45,X, 47,XXX, 47,XXY, Microdeletion, Microduplication	1048	1	0	–	0	1	0	Mixture	11	100

In 2016, Taylor-Phillips et al. [18] reported that the combined PPV of NIPT for screening T21, T18, and T13 was limited to 37–90% and that the PPV for T21 was limited to 45–99%. In our study, the combined PPV of NIPT for screening T21 and T18 was 33.3%, and the PPV for T21 was 50%, which was in general agreement with Taylor-Phillips et al.

Regarding the effectiveness of screening for an abnormal number of sex chromosomes, NIPT is not stable, and the accuracy of detection is controversial. Reiss et al. [19] studied 2851 singleton pregnant women and found that the PPV of NIPT for 45,X screening was only 9% (1/11). Zhang et al. [20] studied 10,275 singleton pregnant women and found that the PPV of NIPT for 45,X screening was 29.41% (5/17). Among the 1048 women with twin pregnancies included in this study, 7 had a high risk of SCA, and the karyotype analysis results showed that the 7 foetal karyotypes were normal. Yang et al. [13] reported NIPT in 432 women with twin pregnancies, with a result showing a high risk for 47,XXX in 1 case, which was proven to be false positive by karyotype analysis. The study data showed that the screening efficacy of NIPT for SCA in twin pregnancy was not satisfactory, especially for 45, X. Its screening efficacy in SCA of twins still needs an extensive amount of study.

For one woman in this study the NIPT results suggested a heterozygous deletion of 6.13 Mb on the long arm of chromosome 15, which was confirmed by karyotype analysis. The study of copy number heterozygosity deficiency in this region shows a definite pathogenic outcome, and the deletion of this region involves Angelman syndrome and Prader-Willi syndrome [21]. Angelman syndrome and Prader-Willi syndrome are characterized by abnormal growth and mental retardation. The pregnant woman underwent a reduction operation at 16 weeks of gestation, and the surviving foetus was well developed after birth. NIPT successfully prevented the pregnant woman from giving birth to a child with birth defects.

In this study, to investigate the clinical application value of NIPT in screening for chromosomal abnormalities in twin pregnancies, 11 false-positive results were obtained. The experimental process was traced to eliminate the possibility of specimen errors. It must be emphasized that NIPT accesses free placental DNA not foetal DNA and placental mosaicism weakens its performance in screening for foetal chromosomal abnormalities [22]. However, it is regrettable that there is no follow-up study on placental cytogenetic characteristics due to various reasons. Therefore, it cannot be absolutely confirmed that the cause of false positives in these cases is restricted to confined placental mosaicism. Studies have shown that NIPT screening cannot completely avoid false-positive results due to confined placental mosaicism, maternal mosaicism, chromosome copy number variation, and maternal tumours, among other confounders [22, 23]. Therefore, when NIPT results are inconsistent with karyotype results, clinicians should be patient in the process of interpretation. We suggest that clinicians should reasonably guide high-risk pregnant women to verify the result by amniotic fluid puncture with the Z value, cffDNA concentration, and B ultrasound.

In this study, 87.5% of the pregnant women were under 35 years of age, and most of them had no previous risk factors. Thus, this study represented the general obstetric population. A number of studies have shown that the success rate of twin primary detection is 94.4% and that the success rate of secondary detection increases to 96.9% [24]. Eight women in this study were tested again after giving consent because the concentration of cffDNA was too low, and the resampling rate was 0.76% (8/1048). In this study, the resampling rate was lower than that reported in the literature, which was related to the detection of not all cases in early pregnancy. The concentration of cffDNA increases with increasing gestational week [25], so the difference in detection time may affect the rate of resampling and failure.

To the best of our knowledge, previous NIPT studies in twin pregnancies have focused mainly on T21, T18, and T13. This study had a large sample size and was the first to evaluate the efficacy of NIPT screening for common trisomy, SCA abnormalities, microdeletion, and microduplication in twin pregnancies. This study provides important data and clinical experience for clinical application. However, the PPV of NIPT is not satisfactory in the screening of chromosomal abnormalities in twin pregnancies, so NIPT is not recommended as a first-line screening technique for aneuploidy diseases of chromosomes in twin pregnancies. NIPT should be used carefully in twin pregnancies. This study did not further clarify the cause of false-positive results and the lack of true-positive data, which are the deficiencies of this study, so the conclusion still needs further confirmation with more positive clinical pregnancy twin data.

## Conclusions

In summary, NIPT based on high-throughput sequencing is a screening technique rather than a diagnostic technique, and its performance in twin pregnancies is weaker than that in singleton pregnancies. However, according to our study, NIPT can detect not only common trisomy but also chromosomal microdeletion in twin pregnancies. Safe and rapid NIPT has certain clinical application value in twin pregnancies, but screening efficiency is unstable. Therefore, it should be used carefully in the screening of chromosomal abnormalities in twins.

## Data Availability

The data used and analysed in the study can be obtained from the corresponding author on reasonable request.
